# Clinical Predictors of Relapse-Free Survival in Early Pulmonary Sarcoidosis

**DOI:** 10.3390/jcm15176875

**Published:** 2026-09-05

**Authors:** Fatma Işıl Uzel, Burak Uzel, Esin Yentürk, Yüksel Peker

**Affiliations:** 1 Department of Pulmonary Medicine, Koc University School of Medicine, 34010 Istanbul, Türkiye; fuzel@kuh.ku.edu.tr; 2 Department of Internal Medicine, Medicana Ataköy Hospital, 34158 Istanbul, Türkiye; burak.uzel41@gmail.com; 3 Department of Pulmonary Medicine, Yedikule Chest Diseases and Surgery Hospital, 34020 Istanbul, Türkiye; eyenturk@gmail.com; 4 Department of Molecular and Clinical Medicine, Sahlgrenska Academy, University of Gothenburg, 40530 Gothenburg, Sweden; 5 Department of Clinical Sciences, Respiratory Medicine and Allergology, Faculty of Medicine, Lund University, 22185 Lund, Sweden; 6 Division of Pulmonary, Allergy and Critical Care Medicine, University of Pittsburgh School of Medicine, Pittsburgh, PA 15213, USA

**Keywords:** sarcoidosis, disease recurrence, radiographic stage, systemic treatment, prognostic factors, long-term follow-up

## Abstract

**Background:** Relapse remains a major challenge in the long-term management of pulmonary sarcoidosis, yet predictors of relapse-free survival in patients with early-stage disease are incompletely understood. We investigated the clinical determinants of time to relapse in a biopsy-confirmed cohort of patients with pulmonary sarcoidosis followed for up to 15 years. **Methods:** This retrospective cohort study included 200 adults (mean age 42.0 ± 11.2 years, 72.0% women) with biopsy-confirmed Stage I–II pulmonary sarcoidosis. The primary outcome was relapse requiring re-initiation or escalation of systemic therapy after remission. Relapse-free survival was evaluated using Kaplan–Meier analysis and compared by the log-rank test. Cox proportional hazards models were used to identify independent predictors of relapse. **Results:** During long-term follow-up, 36 patients (18.0%) experienced disease relapse. Relapse-free survival was significantly lower among patients who received systemic treatment at baseline than among untreated patients (log-rank *p* < 0.001). In multivariable Cox analysis, baseline systemic treatment (adjusted hazard ratio [HR] 4.27, 95% confidence interval [CI] 1.39–13.09; *p* = 0.011) and Scadding Stage II disease (HR 2.58, 95% CI 1.26–5.29; *p* = 0.010) independently predicted relapse. Increasing age was associated with a lower relapse risk (HR 0.70 per 10-year increase, 95% CI 0.50–1.00; *p* = 0.047). Baseline FVC and sex were not independently associated with relapse, while extrapulmonary involvement showed a borderline association (HR 2.16, 95% CI 0.97–4.80; *p* = 0.059). **Conclusions:** Long-term relapse-free survival in early pulmonary sarcoidosis is primarily determined by baseline disease phenotype. Stage II disease identifies patients at increased relapse risk, whereas the association between systemic treatment and relapse most likely reflects confounding by indication rather than a detrimental treatment effect. Time-to-event analysis provides a clinically relevant framework for long-term risk stratification in early-stage sarcoidosis.

## 1. Introduction

Sarcoidosis is a heterogeneous multisystem granulomatous disease of unknown etiology, characterized by the formation of non-caseating granulomas and predominantly affecting the lungs and intrathoracic lymph nodes [1,2]. Its clinical course is highly variable, ranging from spontaneous remission to persistent inflammation, progressive pulmonary fibrosis, and irreversible organ dysfunction [1,2,3]. Although spontaneous remission occurs in many patients with early-stage pulmonary sarcoidosis, a substantial proportion develop recurrent disease requiring repeated courses of systemic therapy, emphasizing the need for reliable predictors of long-term prognosis [2,4].

The decision to initiate systemic treatment remains one of the most challenging aspects of sarcoidosis management. Current international guidelines recommend treatment primarily for patients with clinically significant symptoms, progressive pulmonary function impairment, or potentially irreversible organ involvement rather than radiographic stage alone [4]. More recently, a WASOG position paper proposed a paradigm shift in sarcoidosis management, arguing against routine prolonged corticosteroid therapy and emphasizing individualized treatment strategies [5].

Nevertheless, radiographic staging according to the Scadding classification continues to provide valuable prognostic information, with Stage II disease generally associated with a lower likelihood of spontaneous remission and a greater risk of chronic disease than Stage I [3,4]. However, patients within the same radiographic stage frequently exhibit markedly different clinical trajectories, suggesting that conventional staging alone does not adequately capture disease activity or predict long-term relapse.

Relapse after remission represents one of the major challenges in the long-term management of pulmonary sarcoidosis because it often necessitates prolonged corticosteroid therapy and, in selected patients, steroid-sparing immunosuppressive treatment. Previous studies have reported relapse rates ranging from approximately 20% to 40%, depending on patient selection, treatment strategy, and duration of follow-up [2,6]. A recent systematic review and meta-analysis including 51 studies and 6093 patients reported a pooled relapse prevalence of 39% and identified more advanced radiographic stage as an important predictor of relapse [7]. These findings further support the need for reliable risk stratification tools in patients with pulmonary sarcoidosis.

Several baseline characteristics, including pulmonary function impairment, extrapulmonary organ involvement, and serum angiotensin-converting enzyme (ACE) levels, have been proposed as potential prognostic markers; however, their independent value for predicting relapse remains inconsistent across studies [1,2,8,9].

Most previous studies have evaluated relapse as a binary outcome using logistic regression or have been limited by relatively short follow-up periods [6]. However, relapse is inherently a time-dependent clinical event, and analyses that ignore differences in follow-up duration may not fully characterize long-term disease behavior. Time-to-event approaches, including Kaplan–Meier estimation and Cox proportional hazards regression, provide a more appropriate framework for evaluating relapse-free survival over extended follow-up.

In the present study, the term “early pulmonary sarcoidosis” refers specifically to Scadding Stage I–II radiographic disease and does not denote a predefined duration from symptom onset or diagnosis. Therefore, we investigated long-term relapse-free survival in a biopsy-confirmed cohort of patients with Stage I–II pulmonary sarcoidosis followed for up to 15 years. Using survival analysis and multivariable Cox regression with multiple imputation for missing pulmonary function data, we aimed to identify independent clinical predictors of relapse. We hypothesized that baseline disease phenotype, particularly radiographic stage, rather than systemic treatment itself, would be the principal determinant of long-term relapse risk.

## 2. Materials and Methods

### 2.1. Study Design and Study Population

This retrospective single-center cohort study included consecutive adult patients (≥18 years) with biopsy-confirmed pulmonary sarcoidosis who were evaluated at the Department of Pulmonary Medicine between January 2002 and December 2016. Histopathological confirmation was obtained from all patients using standard diagnostic procedures, including mediastinoscopy, endobronchial ultrasound-guided transbronchial needle aspiration (EBUS-TBNA), transbronchial biopsy, peripheral lymph node biopsy, skin biopsy, and other tissue sampling techniques. For the purposes of this study, “early pulmonary sarcoidosis” was operationally defined as Scadding Stage I–II radiographic disease, irrespective of the duration from symptom onset or diagnosis. Patients with Stage 0 or Stage III disease were excluded because of their very small numbers and substantially different clinical characteristics. A total of 200 patients comprised the final study cohort.

The study was approved by the Institutional Ethics Committee (approval No. 2016/72) and conducted according to the principles of the Declaration of Helsinki. Owing to the retrospective study design, the requirement for informed consent was waived.

### 2.2. Baseline Assessment

Baseline demographic and clinical variables were extracted from electronic medical records, including age, sex, Scadding radiographic stage, pulmonary function tests, extrapulmonary organ involvement, histopathological findings, and treatment status at diagnosis.

Pulmonary sarcoidosis was staged according to the Scadding radiographic classification. Stage I was defined as bilateral hilar lymphadenopathy without pulmonary infiltrates, whereas Stage II was defined as bilateral hilar lymphadenopathy accompanied by pulmonary parenchymal infiltrates.

Systemic treatment consisted primarily of oral methylprednisolone. During the study period, treatment decisions were based on contemporary clinical practice and generally reflected clinically significant symptoms, pulmonary function impairment, persistent disease activity despite observation or inhaled therapy, extrapulmonary organ involvement, or parenchymal lung disease. Initial methylprednisolone doses were generally 40 mg/day, followed by gradual tapering over approximately 6–12 months. Patients requiring relapse treatment could receive steroid-sparing immunosuppressive agents according to clinical judgement. Because treatment decisions were based on the overall clinical phenotype and multiple indications frequently coexisted, a single primary indication for systemic treatment was not prospectively coded. For descriptive purposes, pulmonary function impairment was defined as FVC or FEV_1_ < 80% of the predicted value.

### 2.3. Outcome Definition

The primary outcome was relapse-free survival. Relapse was defined as recurrent clinical and/or radiological disease activity requiring re-initiation or escalation of systemic treatment after a period of remission. Remission was defined as stabilization or resolution of clinical and radiological disease without ongoing systemic treatment. Follow-up time was calculated from the date of diagnosis until the first documented relapse or the date of the last clinical follow-up for patients without relapse.

### 2.4. Statistical Analysis

Continuous variables are presented as mean ± standard deviation (SD) or median (interquartile range), as appropriate, whereas categorical variables are expressed as frequencies and percentages. Baseline characteristics were compared using the independent-samples *t* test or Mann–Whitney *U* test for continuous variables and the χ^2^ test or Fisher’s exact test for categorical variables.

Relapse-free survival was estimated using the Kaplan–Meier method, and survival curves were compared using the log-rank test.

Associations between baseline characteristics and relapse were evaluated using Cox proportional hazards regression. Univariable analyses were initially performed for clinically relevant demographic, radiographic, physiological, and treatment variables. Variables considered clinically important were subsequently entered into multivariable Cox proportional hazards models irrespective of their statistical significance in the univariable analyses.

Baseline FVC and FEV_1_ measurements were unavailable for 29 patients (14.5%). Given the retrospective nature and long inclusion period of the study, specific reasons for missing pulmonary function measurements were not systematically documented. Missingness was considered plausibly related to observed clinical and temporal characteristics rather than to the unobserved pulmonary function values themselves, and a missing-at-random (MAR) assumption was therefore adopted for the primary analysis. Missing pulmonary function variables were handled using multiple imputation by chained equations with predictive mean matching. Twenty imputed datasets were generated, and pooled parameter estimates and standard errors were calculated according to Rubin’s rules. Because the MAR assumption cannot be directly verified, a complete-case analysis was additionally performed as a sensitivity analysis.

Hazard ratios (HRs) with corresponding 95% confidence intervals (CIs) are reported. Two-sided *p* values < 0.05 were considered statistically significant.

All statistical analyses were performed using IBM SPSS Statistics version 28.0 (IBM Corp., Armonk, NY, USA) and R version 4.x (R Foundation for Statistical Computing, Vienna, Austria).

### 2.5. Sensitivity Analyses

A complete-case Cox regression analysis was additionally performed to evaluate the robustness of the findings obtained after multiple imputation. Hazard ratios derived from complete-case and pooled analyses were compared as a sensitivity analysis.

## 3. Results

### 3.1. Study Population

**Figure 1:**
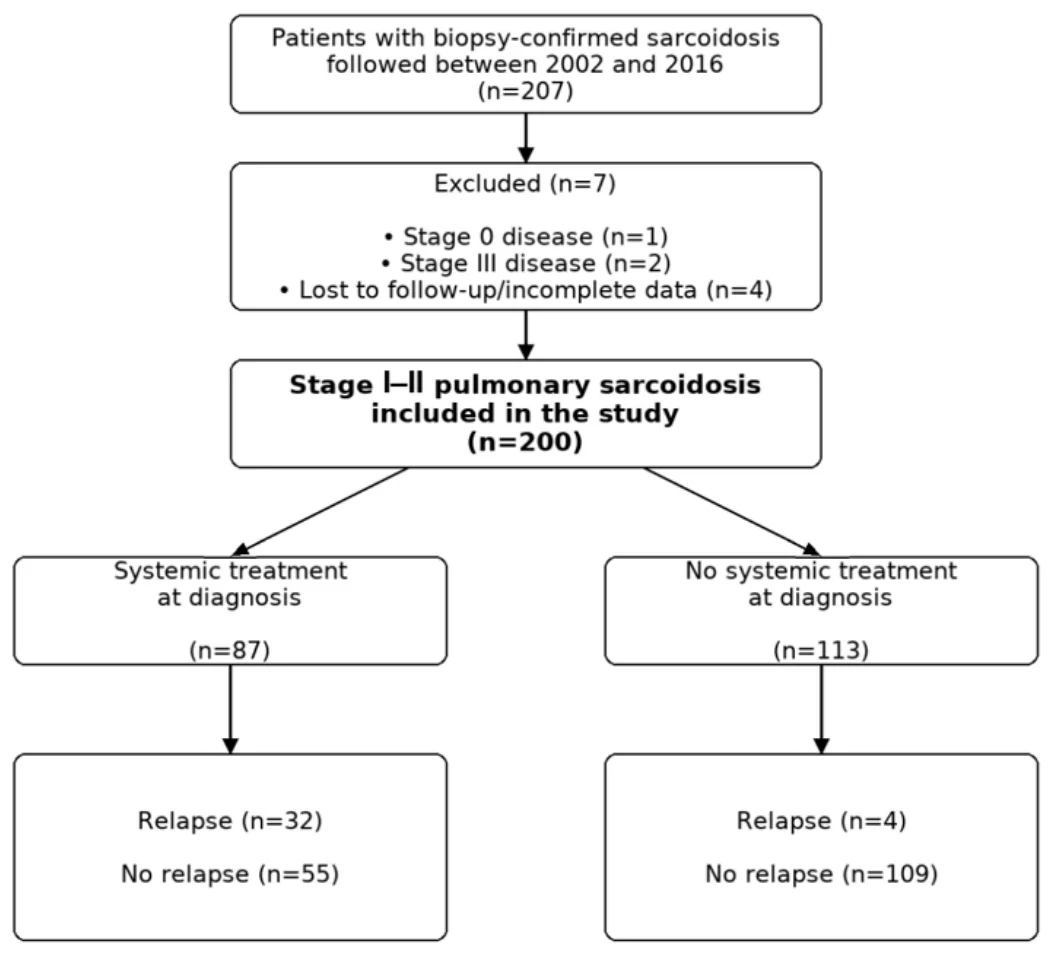
Flowchart of patient selection and study cohort.

As illustrated in Figure 1, the study included 200 patients with biopsy-confirmed Stage I–II pulmonary sarcoidosis, comprising 147 (73.5%) patients with Stage I disease and 53 (26.5%) with Stage II disease.

Baseline demographic and clinical characteristics according to radiographic stage are summarized in Table 1. Patients with Stage II disease were significantly older than those with Stage I disease (44.9 ± 11.6 vs. 40.7 ± 11.0 years, *p* = 0.024). They also had significantly lower baseline pulmonary function, with lower FVC (% predicted) (*p* = 0.003) and FEV_1_ (% predicted) (*p* < 0.001). Extrapulmonary involvement was more frequent in Stage II patients (54.7% vs. 34.7%, *p* = 0.017), who were also more likely to receive systemic treatment at diagnosis (60.4% vs. 37.4%, *p* = 0.006). During follow-up, relapse occurred significantly more often in Stage II than in Stage I disease (35.8% vs. 11.6%, *p* < 0.001). Median follow-up duration was similar between the two groups (*p* = 0.847).

Overall, extrapulmonary involvement was present in 80 patients (40.0%). The most frequently recorded manifestations were peripheral/supraclavicular lymph-node involvement (n = 30), ocular involvement/uveitis (n = 13), cutaneous involvement (n = 12), renal involvement (n = 11), and subcutaneous involvement (n = 8). Less frequent manifestations included sinus (n = 3), thyroid (n = 3), hepatic (n = 2), pericardial (n = 1), and splenic involvement (n = 1). Multiple extrapulmonary sites could be present in the same patient.

### 3.2. Relapse During Follow-Up

During a maximum follow-up of 15 years, 36 patients (18.0%) experienced at least one relapse requiring re-initiation or escalation of systemic therapy. Relapse occurred considerably more frequently among patients receiving systemic treatment at baseline than among untreated patients (32/87 vs. 4/113). Similarly, relapse was more common in Stage II than in Stage I disease.

Among the 87 patients who received systemic treatment at baseline, 71 (81.6%) had respiratory symptoms, 53 (60.9%) had extrapulmonary involvement, and 32 (36.8%) had Scadding Stage II disease. Baseline pulmonary function measurements were available in 79 treated patients; FVC < 80% predicted was present in 30 (38.0%) and FEV_1_ < 80% predicted in 41 (51.9%). These clinical features frequently overlapped, and a single primary indication for systemic treatment could not be retrospectively assigned.

### 3.3. Relapse-Free Survival

**Figure 2:**
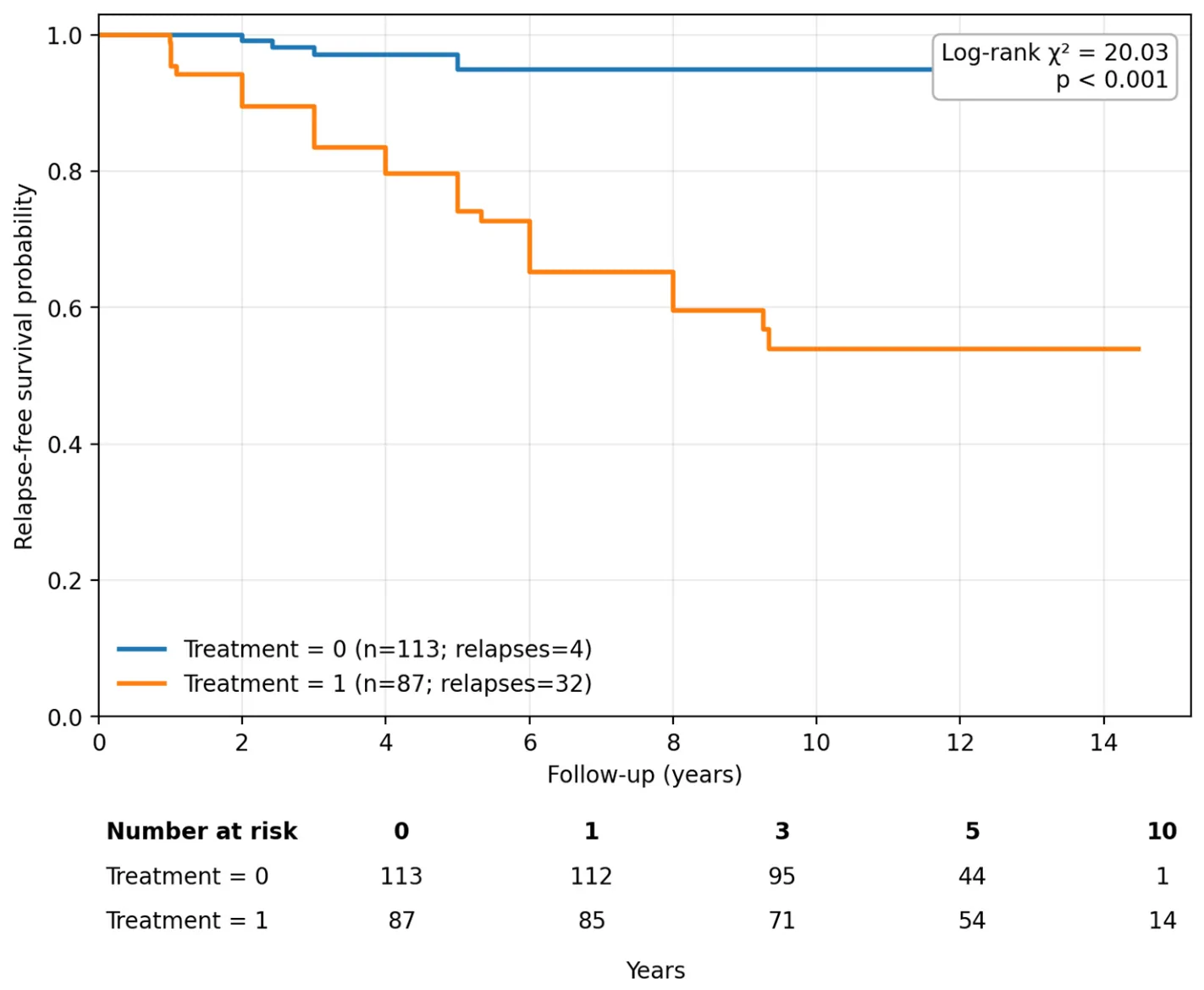
Kaplan–Meier estimates of relapse-free survival according to baseline systemic treatment. Patients who received systemic treatment at diagnosis exhibited significantly lower relapse-free survival than untreated patients during long-term follow-up (log-rank *p* < 0.001). The higher relapse risk observed in treated patients most likely reflects greater baseline disease severity (confounding by indication) rather than a detrimental effect of treatment itself.

Kaplan–Meier analysis demonstrated significantly lower relapse-free survival among patients who received systemic treatment at baseline (log-rank *p* < 0.001) (Figure 2). Separation of the survival curves occurred early during follow-up and persisted throughout the observation period.

**Figure 3:**
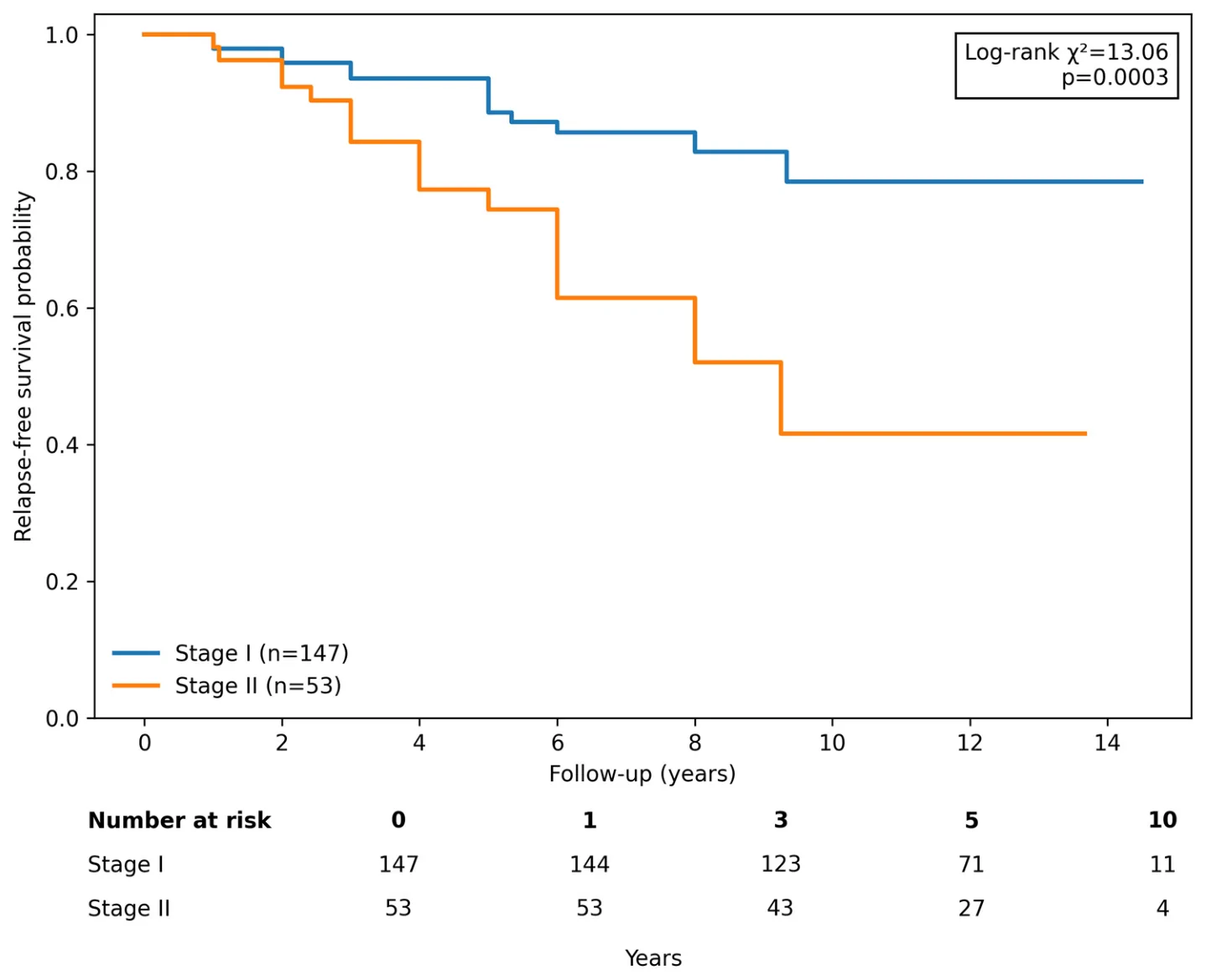
Kaplan–Meier estimates of relapse-free survival according to Scadding radiographic stage. Patients with Stage II pulmonary sarcoidosis exhibited significantly lower relapse-free survival than those with Stage I disease during long-term follow-up (log-rank χ^2^ = 13.06, *p* = 0.0003).

Similarly, patients with Stage II disease exhibited significantly lower relapse-free survival than those with Stage I disease (Figure 3).

### 3.4. Predictors of Relapse

In univariable Cox proportional hazards analyses, baseline systemic treatment, Stage II disease, extrapulmonary involvement, and younger age were associated with relapse-free survival, whereas sex and baseline pulmonary function were not consistently associated with relapse (Table 2A).

As shown in Table 2B, after adjustment for clinically relevant covariates and multiple imputation for missing pulmonary function data, systemic treatment remained independently associated with relapse (adjusted HR 4.27, 95% CI 1.39–13.09; *p* = 0.011). Likewise, Stage II disease independently predicted relapse (adjusted HR 2.58, 95% CI 1.26–5.29; *p* = 0.010). Increasing age was associated with a lower risk of relapse (adjusted HR 0.70 per 10-year increase, 95% CI 0.50–1.00; *p* = 0.047). Neither sex nor baseline FVC independently predicted relapse, whereas extrapulmonary involvement showed a borderline association with relapse risk (adjusted HR 2.16, 95% CI 0.97–4.80; *p* = 0.059).

### 3.5. Sensitivity Analysis

Sensitivity analysis using complete-case data yielded findings consistent with the primary multiple-imputation analysis. Baseline systemic treatment remained independently associated with relapse (adjusted HR 3.43, 95% CI 1.10–10.68; *p* = 0.033), as did Scadding Stage II disease (adjusted HR 2.68, 95% CI 1.25–5.73; *p* = 0.011). Age showed a similar inverse association with relapse (adjusted HR 0.72 per 10-year increase, 95% CI 0.50–1.03; *p* = 0.072), whereas sex, baseline FVC, and extrapulmonary involvement were not independently associated with relapse.

## 4. Discussion

The present study evaluated long-term relapse-free survival in a biopsy-confirmed cohort of patients with Stage I–II pulmonary sarcoidosis followed for up to 15 years. Using survival analyses with Cox proportional hazards regression, we identified two principal findings. First, patients with Stage II disease experienced significantly lower relapse-free survival than those with Stage I disease. Second, although baseline systemic treatment was independently associated with subsequent relapse, this association should be interpreted cautiously because treatment most likely identified patients with a more severe clinical phenotype rather than representing a causal determinant of disease recurrence. These findings support a phenotype-based approach to long-term risk stratification in early pulmonary sarcoidosis [1,2,4].

The overall relapse rate in our cohort was 18.0%, which is lower than the pooled prevalence of 39% reported in a recent systematic review and meta-analysis [7]. This difference likely reflects, at least in part, differences in study populations and outcome definitions. Our cohort was restricted to Scadding Stage I–II disease, thereby excluding patients with more advanced radiographic involvement who may have a greater propensity for recurrent or persistent disease. In addition, we applied a clinically stringent definition of relapse, requiring recurrent clinical and/or radiological disease activity that prompted re-initiation or escalation of systemic therapy after remission. Thus, mild clinical or isolated radiological recurrence not requiring systemic treatment was not counted as an event. Heterogeneity in patient selection, relapse definitions, treatment strategies, and follow-up duration across previous studies may further explain the higher pooled relapse estimate [7].

Radiographic stage remains one of the oldest and most widely used prognostic tools in pulmonary sarcoidosis. More than six decades after the original observations by Scadding, parenchymal lung involvement continues to identify patients with a lower probability of spontaneous remission and a greater likelihood of persistent inflammatory disease [3]. Our Kaplan–Meier analyses extend these observations by demonstrating not only a higher relapse frequency but also significantly shorter relapse-free survival among patients with Stage II disease. Our findings are consistent with a recent meta-analysis demonstrating that advanced radiographic stages are associated with a significantly higher risk of relapse compared with Stage I disease [7]. This finding is also consistent with contemporary ERS recommendations and recent reviews emphasizing that pulmonary parenchymal involvement reflects a greater inflammatory burden requiring closer longitudinal monitoring [1,2,4]. In clinical practice, radiographic stage remains a simple and readily available marker that can assist clinicians in identifying patients who may benefit from more intensive follow-up after remission.

One of the most interesting findings was the strong association between baseline systemic treatment and subsequent relapse. At first glance, this observation could suggest that corticosteroid treatment predisposes patients to recurrent disease. However, such an interpretation would be misleading. Current treatment recommendations advocate systemic therapy only for patients with clinically significant symptoms, progressive pulmonary impairment, or major extrapulmonary involvement [4]. More recently, a WASOG position paper further emphasized that treatment decisions should be individualized according to disease severity, symptom burden, and risk of irreversible organ damage, while discouraging prolonged maintenance corticosteroid therapy whenever possible [5]. Consequently, patients receiving treatment generally represent a subgroup with greater disease activity at baseline. This observation is a classic example of confounding by indication, whereby patients selected for treatment inherently have more active or severe disease than those managed conservatively. The attenuation of the treatment-associated hazard after adjustment for baseline clinical characteristics further supports the likelihood of confounding by indication. Therefore, systemic treatment in this study should be viewed primarily as a surrogate marker of baseline disease severity rather than an independent biological cause of relapse. Similar observations have been reported in previous cohort studies, in which relapse occurred more frequently among treated patients because treatment was preferentially initiated in patients with more extensive or active disease [2,6].

Baseline pulmonary function has been proposed as an important prognostic indicator in sarcoidosis. Stage II patients in our cohort had significantly lower baseline FVC, reflecting greater pulmonary involvement. Nevertheless, after adjustment for radiographic stage and other clinically relevant variables, FVC was not independently associated with relapse-free survival. This finding suggests that pulmonary function impairment may largely reflect the extent of parenchymal disease rather than providing additional prognostic information beyond radiographic phenotype. Our results are broadly consistent with previous studies showing that although preserved FVC is associated with better overall outcomes, its independent ability to predict relapse remains limited [8].

Extrapulmonary involvement showed a borderline independent association with relapse risk. Although this association did not reach conventional statistical significance, the magnitude of the estimated hazard ratio suggests that multisystem disease may identify patients with a more active inflammatory phenotype. Importantly, extrapulmonary involvement in our cohort was heterogeneous and predominantly comprised peripheral lymph-node, ocular, cutaneous, renal, and subcutaneous manifestations. The small numbers within individual organ categories precluded meaningful organ-specific survival analyses; therefore, the borderline association observed for extrapulmonary involvement should be interpreted as reflecting overall multisystem disease burden rather than the prognostic effect of any particular organ manifestation. Previous Turkish and international cohorts have likewise demonstrated that extrapulmonary manifestations frequently coexist with more severe thoracic disease and contribute to overall disease burden [10,11]. Larger prospective studies are needed to clarify whether extrapulmonary involvement independently predicts long-term relapse.

An additional strength of the present study is the use of survival analysis. Most previous investigations have analysed relapse as a dichotomous outcome, thereby ignoring differences in follow-up duration between patients [6]. Because relapse is inherently a time-dependent event, Kaplan–Meier estimation and Cox proportional hazards modelling provide a more clinically meaningful assessment of disease behaviour over prolonged follow-up. Furthermore, missing pulmonary function data were addressed using multiple imputation, reducing potential bias associated with complete-case analyses and improving the robustness of the multivariable models.

The findings of the present study have several potential clinical implications. Patients with Stage II pulmonary sarcoidosis had substantially shorter relapse-free survival than those with Stage I disease, suggesting that radiographic stage may remain useful as a simple component of post-remission risk assessment. Patients with parenchymal involvement, particularly when accompanied by extrapulmonary disease or a previous requirement for systemic treatment, may therefore warrant closer clinical surveillance after treatment withdrawal or remission. Importantly, however, our findings should not be interpreted as supporting treatment decisions based on radiographic stage alone. Rather, they reinforce a phenotype-based approach in which radiographic findings are considered together with symptoms, pulmonary function, extrapulmonary manifestations, and previous treatment requirements. Similarly, the higher relapse risk observed among previously treated patients should not be interpreted as evidence that systemic treatment promotes relapse but rather as a marker of a clinical phenotype with greater underlying disease activity. Recent cohort data have additionally suggested that smoking history and prolonged corticosteroid exposure may contribute to recurrence risk, highlighting the multifactorial nature of relapse in sarcoidosis [12].

Future prospective multicenter studies should validate these findings in larger and more diverse sarcoidosis populations and determine whether combinations of readily available clinical variables can provide greater prognostic discrimination than individual predictors. Such studies should prospectively record treatment indications, cumulative corticosteroid exposure, steroid-sparing therapies, organ-specific extrapulmonary involvement, and standardized definitions and timing of remission and relapse. Integration of these clinical features with emerging imaging, physiological, and biomarker-based measures may ultimately permit development of validated relapse-risk models. If externally validated, such models could support risk-adapted follow-up strategies, with more intensive surveillance for patients at higher risk while potentially reducing unnecessary long-term monitoring in patients with a favorable phenotype. The present study has several strengths, including a relatively large cohort of biopsy-confirmed patients, long-term follow-up extending up to 15 years, comprehensive clinical characterization, and the application of contemporary survival analyses with multiple imputation for missing pulmonary function data. These methodological features increase the robustness of our findings and distinguish this study from previous retrospective analyses that relied primarily on binary outcome models.

Several limitations should also be acknowledged. First, this was a retrospective single-center study, limiting generalizability. Second, treatment allocation was not randomized, and residual confounding by indication cannot be completely excluded despite multivariable adjustment. In addition, the primary clinical indication for systemic treatment was not prospectively coded, precluding a definitive attribution of treatment initiation to a single clinical feature. Third, detailed information regarding cumulative corticosteroid exposure, tapering schedules, and steroid-sparing immunosuppressive therapy was not uniformly available. Finally, the relatively small number of relapse events limited the inclusion of additional variables in the multivariable model and warrants confirmation in larger prospective multicenter cohorts.

## 5. Conclusions

In conclusion, long-term relapse-free survival in early pulmonary sarcoidosis is primarily determined by baseline disease phenotype. Stage II disease independently predicts relapse, whereas the association between systemic treatment and relapse most likely reflects greater disease severity at treatment initiation rather than a harmful effect of therapy. Survival analysis provides a clinically relevant framework for long-term prognostic assessment and may facilitate more individualized follow-up strategies in patients with early pulmonary sarcoidosis.

## Figures and Tables

**Table 1:** Baseline demographic and clinical characteristics according to Scadding stage.

Variable	Stage I (n = 147)	Stage II (n = 53)	*p* Value
Age, years	40.7 ± 11.0	44.9 ± 11.6	0.024
Female sex, n (%)	108 (73.5%)	36 (67.9%)	0.53
FVC, % predicted ^†^	91.4 ± 15.7	82.8 ± 17.2	0.003
FEV_1_, % predicted ^†^	89.7 ± 16.8	76.8 ± 19.5	<0.001
Extrapulmonary involvement, n (%)	51 (34.7%)	29 (54.7%)	0.017
Systemic treatment, n (%)	55 (37.4%)	32 (60.4%)	0.006
Relapse during follow-up, n (%)	17 (11.6%)	19 (35.8%)	<0.001
Follow-up duration, years ^‡^	5.0 (3.5–7.7)	5.2 (3.9–8.0)	0.847

Data are presented as mean ± standard deviation, median (interquartile range), or number (%). ^†^ Missing pulmonary function data were handled using multiple imputation for the multivariable analyses. ^‡^ Median (interquartile range). Abbreviations: FEV_1_, forced expiratory volume in one second; FVC, forced vital capacity; IQR, interquartile range; SD, standard deviation.

**Table 2:** (**A**) Univariable Cox proportional hazards analyses for relapse-free survival. (**B**) Multivariable Cox proportional hazards analysis for relapse-free survival (primary model).

**(A)**			
**Variable**	**Hazard Ratio (HR)**	**95% CI**	***p*** **Value**
Age (per 10-year increase)	0.76	0.56–1.03	0.079
Male sex	1.14	0.54–2.42	0.73
Scadding Stage II (vs. Stage I)	3.08	1.59–5.95	<0.001
Systemic treatment	7.46	2.66–20.95	<0.001
FVC (% predicted, per 10% increase)	0.91	0.73–1.14	0.41
Extrapulmonary involvement	2.32	1.11–4.82	0.025
**(B)**			
**Variable**	**Adjusted HR**	**95% CI**	***p*** **Value**
Age (per 10-year increase)	0.70	0.50–1.00	0.047
Male sex	1.19	0.55–2.58	0.663
Scadding Stage II (vs. Stage I)	2.58	1.26–5.29	0.010
Systemic treatment	4.27	1.39–13.09	0.011
FVC (% predicted, per 10% increase)	1.05	0.82–1.35	0.678
Extrapulmonary involvement	2.16	0.97–4.80	0.059

Abbreviations: CI, confidence interval; HR, hazard ratio; FVC, forced vital capacity. Model: Multivariable Cox proportional hazards regression adjusted for age, sex, Scadding stage, systemic treatment, baseline FVC (% predicted), and extrapulmonary involvement. Missing pulmonary function data were handled using multiple imputation by chained equations (20 imputations), and pooled estimates were calculated according to Rubin’s rules. Scadding Stage II was defined by the presence of pulmonary parenchymal infiltrates in addition to bilateral hilar lymphadenopathy; therefore, parenchymal involvement was not entered separately into multivariable models because of collinearity.

## Data Availability

Data collected for the study, including de-identified individual participant data, will be made available to others within 6 months of the publication of this article for academic purposes (e.g., meta-analyses) upon request to the corresponding author (yuksel.peker@lungall.gu.se).

## Abbreviations

The following abbreviations are used in this manuscript:

ACE	Angiotensin-converting enzyme
CI	Confidence interval
EBUS-TBNA	Endobronchial ultrasound-guided transbronchial needle aspiration
ERS	European Respiratory Society
FEV_1_	Forced expiratory volume in one second
FVC	Forced vital capacity
HR	Hazard ratio
IQR	Interquartile range
MAR	Missing at random
SD	Standard deviation
WASOG	World Association of Sarcoidosis and Other Granulomatous Disorders

## References

[1] Drent M., Crouser E.D., Grunewald J. (2021). Challenges of sarcoidosis and its management. N. Engl. J. Med..

[2] Belperio J.A., Shaikh F., Abtin F.G., Fishbein M.C., Weigt S.S., Saggar R., Lynch J.P. (2022). Diagnosis and treatment of pulmonary sarcoidosis: A review. JAMA.

[3] Scadding J.G. (1961). Prognosis of intrathoracic sarcoidosis in England: A review of 136 cases after five years’ observation. Br. Med. J..

[4] Baughman R.P., Valeyre D., Korsten P., Mathioudakis A.G., Wuyts W.A., Wells A., Rottoli P., Nunes H., Lower E.E., Judson M.A. (2021). ERS clinical practice guidelines on treatment of sarcoidosis. Eur. Respir. J..

[5] Wells A.U., Lower E.E., Baughman R.P., Culver D.A., Judson M.A., Bonham C.A., Gerke A.K., Grutters J.C., Knoet C., Martone F. (2025). A paradigm shift in corticosteroid therapy for sarcoidosis: A World Association of Sarcoidosis and Other Granulomatous Disorders Position Paper, endorsed by the Americas Association of Sarcoidosis and Other Granulomatous Disorders. Lancet Respir. Med..

[6] Murata O., Suzuki K., Takeuchi T., Kudo A. (2021). Incidence and baseline characteristics of relapse or exacerbation in patients with pulmonary sarcoidosis in Japan. Sarcoidosis Vasc. Diffus. Lung Dis..

[7] Hashim Z., Baughman R.P., Agarwal R., Tripathy N.K., Gupta M., Khan A., Kumar A., Kumar A., Nath A. (2026). Prevalence of relapse in pulmonary sarcoidosis: A systematic review and meta-analysis. Respir. Med..

[8] Judson M.A., Yucel R., Preston S., Chen E.S., Culver D.A., Hamzeh N., Lower E.E., Sweiss N.J., Valeyre D., Veltkamp M. (2022). The association of baseline sarcoidosis measurements with 6-month outcomes that are of interest to patients: Results from the On-line Sarcoidosis Assessment Platform Study (OSAP). Respir. Med..

[9] Ungprasert P., Carmona E.M., Utz J.P., Ryu J.H., Crowson C.S., Matteson E.L. (2016). Diagnostic utility of angiotensin-converting enzyme in sarcoidosis: A population-based study. Lung.

[10] Kiter G., Müsellim B., Çetinkaya E., Türker H., Kunt Uzaslan A.E., Yentürk E., Uzun O., Saǧlam L., Özdemir Kumbasar O., Çelik G. (2011). Clinical presentations and diagnostic work-up in sarcoidosis: A series of Turkish cases. Tuberc. Thorac..

[11] Okumus G., Musellim B., Cetinkaya E., Turker H., Uzaslan E., Yenturk E., Uzun O., Saglam L., Kumbasar O.O., Celik G. (2011). Extrapulmonary involvement in patients with sarcoidosis in Turkey. Respirology.

[12] Bertuccio F., Piloni D., Crescenzi M., Tousa F., Putignano P., Russo M., Maragò I., Corsico A.G., Stella G.M. (2026). Sarcoidosis relapse risk factors: A single-centre retrospective cluster analysis. BMJ Open Respir. Res..

